# High seroprevalence of syphilis infection among pregnant women in Yiregalem hospital southern Ethiopia

**DOI:** 10.1186/s12879-018-2998-8

**Published:** 2018-03-06

**Authors:** Anteneh Amsalu, Getachew Ferede, Demissie Assegu

**Affiliations:** 1 Department of Medical Microbiology, University of Gondar, P.O.Box 196, Gondar, Ethiopia; 2 Department of Medical Laboratory Sciences, Hawassa University, Hawassa, Ethiopia

**Keywords:** Syphilis, Pregnant women, Ethiopia

## Abstract

**Background:**

Despite availability of effective treatment and the implementation of focused antenatal care (ANC), still the prevalence of syphilis persists in Ethiopia. Yet, data is not found in southern Ethiopia. Therefore, this study was conducted to determine the seroprevalence and associated factors of syphilis among pregnant women in Yiregalem Hospital, Southern Ethiopia.

**Methods:**

A cross-sectional study was conducted among pregnant women from October 2015 to August 2016. Data on socio-demography and obstetric conditions of pregnant women were collected using a structured questionnaire. Serum samples were screened for syphilis using rapid plasma regain (RPR) test and those found seropositive were further confirmed by *Treponema pallidum* haemagglutination assay (TPHA) test following the manufacturer’s instruction. HIV results were reviewed from records. The data were analyzed using SPSS version 20 software.

**Results:**

Of 494 pregnant women, 204(41.3%) were first ANC visit attenders. Of these, 123(60.3%) were after the 12th gestational week. Sero-prevalence of syphilis and HIV was found to be 5.1% (25/494; 95 CI, 3.2-7.1%) and 10.3% (51/494; 95 CI, 7.7-13.2%), respectively. The overall prevalence of syphilis and HIV co-infection was 2.2% (11/494). Women with HIV infection were significantly more likely to be syphilis-seropositive (AOR = 10.3, 95%CI, 4.213-25.185) than HIV negatives.

**Conclusions:**

High seroprevalence of syphilis particularly among HIV positive women and late first ANC visit attenders in the study area calls for further ramping up of current intervention measures. Like integration of syphilis testing and treatment to the already established HIV prevention program and creating awareness about early ANC visit and follow-up.

## Background

Despite, widely available and affordable screening methods and treatment, syphilis remains a public health problem in pregnant women worldwide particularly in sub-Saharan Africa (SSA) [1, 2]. An estimated 2.7% (0.1–10.3%) of pregnant women in SSA are infected with syphilis, representing more than 900,000 pregnancies at risk each year [1]. In Ethiopia, according to ANC-based sentinel surveillances, syphilis prevalence increased from 1%, in 2012 to 1.2%, in 2014 [3] and as high as 2.9 to 3.7% in recent studies among ANC attendees in Gondar [4–6] and 7.3 to 9.8% among HIV patients in Hawassa [7] and Addis Ababa [8], respectively.

Syphilis is a chronic infectious disease caused by the Spirochaete, *Treponema pallidum.* It causes ulcerative genital lesion that enhances the acquisition and sexual transmission of HIV infection [2]. It has been shown that maternal syphilis can increase HIV transmission during sexual intercourse, in utero or at delivery by two- to- fivefold [9, 10]. Patients with concurrent HIV infection are thought to be at increased risk of neurological complications and treatment failure [11]. Kuznik et al. reported an estimate of 205,901 adverse pregnancy outcomes, such as spontaneous abortion, stillbirth, low birth weight, neonatal death and congenital syphilis, due to untreated maternal syphilis [12]. Ethiopia is among the three SSA countries with the highest numbers of adverse pregnancy outcomes [12]. Due to these adverse outcomes.

The World Health Organization (WHO) recommends that the first antenatal visit should be initiated at ≤12 weeks and at least four ANC visits are required at specified intervals for healthy pregnant women with no underlying medical problems, in focused antenatal care [13]. Importantly, control of vertical transmission of syphilis and associated birth complications is possible through timely screening of all pregnant mothers at the first prenatal visit and those who are at high risk for syphilis, are previously untested, or live in areas of high syphilis prevalence should be screened again early in the third trimester and at delivery and treated if positive [14, 15]. However, in practice, there is considerable screening and therapeutic heterogeneity. In Ethiopia, pregnant women are tested only once for syphilis during ANC visits using rapid plasma reagin (RPR) test and those women reactive for RPR were treated immediately with one or more dose of intramuscular benzathine penicillin G 2.4 million units [16]. However, the burden of this disease in pregnancy persists [5, 6] and the burden may vary depending on the variation in the timing and frequency of ANC visit, diagnosis and treatment, socio-demographic variables, cultural practice and socio-economic factors [17]. Such data is scarce in Ethiopia [5, 6], particularly in the study area. Therefore, this study was aimed to provide information for health policy makers to plan effective strategies to control syphilis transmission during pregnancy.

## Methods

### Study design, area and period

A cross sectional study was conducted among pregnant women attending in Yirgalem hospital ANC clinic from October 2015 to August 2016. The hospital was located 70 km from Hawassa, the capital city of Southern Nations and Nationalities People’s Region (SNNPR) and 273 km from Addis Ababa, the capital city of Ethiopia. It is the largest hospital in Southern Ethiopia and provides medical education and training in addition to medical care. The hospital ANC clinic gives prenatal, delivery and postnatal care services for pregnant women and has 69 bed rooms for pregnant women. HIV and syphilis screening (RPR test only) for pregnant womenis offered at the time of their first ANC visit in the hospital.

### Population

The study population consisted of pregnant women attending ANC clinic in Yirgalem hospital during the study period. Women less than 18 years of age, on treatment for syphilis and labor or delivery during recruitment were excluded. Sample size was estimated to be 342 using single population proportion formula, assuming 3.7% syphilis prevalence in pregnant women in Gondar, Ethiopia [5]; 2% precision and 95% level of confidence. But, in attempting to enhance the statistical power of detecting the rate difference by exposure status, we investigated a total of 500 pregnant women, prospectively. Study populations were selected using systematic random sampling techniques; the first one pregnant woman was selected randomly out of 5 women visiting the hospital and then every 5th women was enrolled in the study until the total sample was achieved. Code number was written with special mark on their follow-up chart to avoid duplication during the study period.

### Data collection

#### Socio-demographic data

After providing written informed consent, participants answered a questionnaire that included questions about their socio-demography and obstetric history. In addition, HIV result and antiretroviral therapy (ART) status were also obtained from their follow-up chart by two midwife nurses.

### Laboratory testing

Five milliliter of blood samples were collected and screened for syphilis using rapid plasma reagin (RPR) test (Human, Germany). Sera tested positive by RPR tests were confirmed by modified *Treponema pallidum* haemagglutination assay (TPHA) (Syphicheck–WB, Qualpro Diagnostics, India). Laboratory tests were carried out according to the instructions of the manufacturers and all tests were run against the positive and negative controls. Only those samples positive by both RPR and TPHA were considered as probable active syphilis (PAS) infection [18].

### Data analysis

Data were coded, entered and analyzed using SPSS version 20(IBM Corp., Armonk, NY, USA). We described data using either proportion or mean with standard deviation (SD). The association between syphilis infection and socio-demographic factors were assessed using crude odds ratio (COR) from a binary logistic regression analysis. Adjusted odds ratio (AOR) was also computed using multivariable logistic regression analysis, taking all factors yielding a *p*-value ≤0.2 in bivariate analysis. A *p*-value of < 0.05 was accepted as significant.

### Ethical consideration

This study was reviewed and approved by the Institutional Review Board (IRB) of Hawassa University College of Medicine and Health Science. A letter of support was obtained from the SNNPR health bureau and Yirgalem hospital administration. Informed written consent was obtained from the study participants and any information obtained during the study was kept with utmost confidentiality, and those found infected with syphilis were treated.

## Results

### Socio-demographic characteristics of study population

Out of the 500 pregnant women, 494 (98.8%) were enrolled in the study, while the rest 6(1.2%) were excluded from the analysis due to incomplete questionnaire. The mean age of pregnant women was 26.5 years (standard deviation [SD], 4.6; range, 18-42 years). Majority 447(90.5%) of study participants were married and had at least primary education 435(88.1%). Greater than two thirds (67.4%) of the pregnant women were urban residents and 246(49.8%) were housewives. Their previous obstetric history revealed a mean of 2.4 gravida (ranges from 1 to 9) and 334(67.6%) of women were multigravida, 239(71.6%) had history of institutional delivery (Table 1).

**Table 1:** Socio-demography and some obstetric conditions of pregnant women at Yirgalem hospital, Southern Ethiopia, 2016

Characteristics		Number (*n* = 494)	Percent (%)
Age(years)	< 25	161	32.6
	25-29	214	43.3
	≥30	119	24.1
Residence	Urban	333	67.4
	Rural	161	32.6
Education	Illiterate	59	11.9
	Primary	168	34.0
	Secondary	166	33.6
	Tertiary	101	20.4
Occupation	House wife	246	49.8
	Health worker	19	3.8
	Employed	133	26.9
	Merchant	52	10.5
	Student	44	8.9
Marital status	Married	447	90.5
	Single/divorced/widowed	47	9.5
Gravidity	Primigravida	160	32.4
	Multigravida(≥2)	334	67.6
Place of previous birth	Home	95	28.4
(excludes primigravida)	Health institution	239	71.6

### Gestational age with frequency of antenatal care visit

Out of 494 women who attended ANC during the study period, 204(41.3%) were first visit attendees. Of these, only 81(39.7%) were in the first trimester while 123(60.3%) were late (in the second and third trimester) attendees. Considering, gestational age the majority (43.2%) of women were in the third trimester. But, only 73(34.1%) out of 214 ANC attenders in the third trimester had attended at least four ANC visits (Table 2).

**Table 2:** The total number of ANC visits and gestational age at the final screening of pregnant women inYirgalem hospital, Southern Ethiopia, 2016

Total number of ANC visits	Gestational age at final screening			
	1^st^ trimester n (%)	2^nd^ trimester n (%)	3^rd^ trimester n (%)	Total n (%)
1	81 (39.7)	79 (38.7)	44 (21.6)	204 (41.3)
2	14 (12.6)	64 (57.7)	33 (29.7)	111 (22.5)
3	5 (5.1)	30 (30.3)	64 (64.6)	99 (20.0)
≥4	0	7 (8.8)	73 (91.2)	80 (16.2)
Total	100 (20.2)	180 (36.5)	214 (43.3)	494 (100)

*ANC* antenatal care

### Seroprevalence of syphilis

Of 494 pregnant women, the prevalence of confirmed syphilis seropositive was 5.1% (25/494; 95 CI, 3.2-7.1%). Twenty-five among 30 participants (6.1%) with a positive RPR were also positive for TPHA. The overall HIV prevalence was found to be 10.3% (51/494; 95 CI, 7.7 -13.2%). Among them, sixteen (31.4%) women knew their HIV status for the first time while, 68.6% (35/51) they knew their HIV status before the study period and had started ART. The overall prevalence of syphilis-HIV co-infection was 2.2% (11/494) (Table 3).

**Table 3:** Syphilis serological tests in pregnant women by HIV status at Yirgalem hospital, Southern Ethiopia, 2016

Syphilis test	Total tested	n (%) syphilis positive	n (%) HIV positive (*N* = 51/494)
RPR	494	30 (6.1)	14 (2.8)
TPHA	30	25 (83.3)	11 (36.7)
Syphilis seropositivity	494	25 (5.1)	11 (2.2)

*HIV* human immunodeficiency virus, *n* total number of syphilis positive, *N* total number of HIV positive, *RPR* rapid plasma regain, *TPHA Treponema pallidum* haemagglutination assay

### Associated factors of syphilis infection

Syphilis prevalence increased with increasing age, with the highest rate in the age ≥ 30 years (5.9%). Similarly, seroprevalence of syphilis increased with gestational period, peak in third trimester (6.1%) and also in women who were occupationally health worker (10.5%), followed by housewives (6.1%), rural resident(6.3%), not cohabiting with their partner (8.5%), multigravidae (5.4%), had more than one ANC visit (5.9%), multiple sexual partner(8.9%), previous history of home delivery(6.3%) and HIV positive (21.6%)(Table 4).

**Table 4:** Associated factors of syphilis infection among pregnant women at Yirgalem hospital, Southern Ethiopia, 2016

Variables		Syphilis		COR (95% CI)	AOR (95%CI)	*P*-value
		NR(*n* = 469)	R(*n* = 25)			
Age(years)	< 25	155 (96.3)	6 (3.7)	1		
	25-29	202 (94.4)	12 (5.6)	1.5 (0.563-4.180)		
	≥30	112 (94.1)	7 (5.9)	1.6 (0.528-4.935)		
Residence	Urban	312 (93.7)	21 (6.3)	2.6 (0.892-7.829)**	2.6 (0.845-7.982)	0.096
	Rural	157 (97.5)	4 (2.5)	1		
Education	Illiterate	57 (96.6)	2 (3.4)	1		
	Elementary	160 (95.2)	8 (4.8)	1.4 (0.294-6.909)		
	Secondary	156 (94.0)	10 (6.0)	1.8 (0.388-8.592)		
	Tertiary	96 (95.0)	5 (5.0)	1.5 (0.279-7.903)		
Occupational	House wife	231 (93.9)	15 (6.1)	2.8 (0.359-21.695)		
	Health worker	17 (89.5)	2 (10.5)	5.0 (0.430-59.525)		
	Employed	128 (96.2)	5 (3.8)	1.7 (0.191-14.780)		
	Merchant	50 (96.2)	2 (3.8)	1.7 (0.151-19.632)		
	Student	43 (97.7)	1 (2.3)	1		
Marital status	Married	426 (95.3)	21 (4.7)	1		
	Not cohabiting*	43 (91.5)	4 (8.5)	1.9 (0.619-5.751)		
Gestational age at screening	First trimester	98 (98.0)	2 (2.0)	1		
	Second trimester	170 (94.4)	10 (5.6)	2.9 (0.619-13.423)**	3.8 (0.778-18.847)	0.099
	Third trimester	201 (93.9)	13 (6.1)	3.2 (0.701-14.319)**	4.6 (0.959-22.340)	0.056
Gravidity	Primigravidae	153 (95.6)	7 (4.4)	1		
	Multigravidae(≥2)	316 (94.6)	18 (5.4)	1.2 (0.509-3.044)		
Frequency of	1	196 (96.1)	8 (3.9)	1		
ANC visit	≥2	273 (94.1)	17 (5.9)	1.5 (0.646-3.606)		
Multiple sexual	Yes	41 (91.1)	4 (8.9)	2.0 (0.651-6.701)		
Partner	No	428 (95.3)	21 (4.7)	1		
Place of	Home	89 (93.7)	6 (6.3)	1.3 (0.464-3.502)		
previous birth	Health institution	227 (95.0)	12 (5.0)	1		
HIV	Non-reactive	429 (96.8)	14 (3.2)	1		
	Reactive	40 (79.4)	11 (21.6)	8.4 (3.859-19.786)**	10.3 (4.213-25.185)	0.001

*Not cohabiting, **single/divorced /widowed; *p*-value < 0.2; *R* reactive, *NR* nonreactive, *COR* crude odds ratio, *AOR* adjusted odds ratio

In multivariate logistic regression analysis, women with HIV infection were significantly more likely to be syphilis-seropositive (AOR =10.3, 95%CI, 4.213-25.185, *p* < 0.001) as compared to HIV negatives. However, age, residence, occupation, marital status, total number of ANC visit, gravidity, multiple sexual factors and previous place of birth were not statistical significant (Table 4).

## Discussion

The seroprevalence of syphilis among pregnant women in this study was 5.1%, which is comparable to the studies conducted in Gondar, Ethiopia (3.7%) [5] and Uganda (5.1%) [19]. In contrast, it is higher than that found in the national sentinel surveillance studies in 2012(1%) and 2014 (1.2%) [3] and previous studies in Gondar, Ethiopia (1-2.9%) [4, 6, 20, 21], Tanzania (1.6-2.5%) [22, 23] and Madagascar (3%) [24]. Moreover, higher seroprevalence of syphilis was also reported among ANC attendees in Tanzania(7.2%) [25], and Zambia(8.2%) [26] and in HIV patients in different parts of Ethiopia(7.3-9.8%) [7, 8]. The observed differences might be due to differences in geographic sites and time-period, the data source (primary or secondary data), socio-cultural and economic factors, and differential access to syphilis diagnosis and treatment.

In this study, syphilis seroprevalence increased slightly with increasing age which is in agreement with the studies conducted elsewhere [5, 7, 24, 27] . This is perhaps due to the risk of exposure to syphilis increased with time. The preponderance of syphilis among women not cohabiting with their partner and urban residence in our study was concordant with findings reported in Gondar [4]. In contrast, high burden of syphilis in the rural area has been reported in the studies from Gondar, Ethiopia [6] and Tanzania [22]. The high burden of syphilis in the urban residents may be partially explained by 68.6% of HIV positive women in our study were urban dwellers as those infections share the same risk factor and one facilitate the presence of the other; as well as commercial sexual practice is relatively more permissible in urban than rural population [28].

In this study, high prevalence of HIV infection (10.3%) among pregnant women attending the ANC was consistent with the previous studies in Gondar (9.6-11.9%) [5, 6, 20, 21]. Women with HIV infection were significantly more likely to be syphilis-seropositive than HIV negatives concordant with the national sentential surveillance [3] and other studies [29, 30]. The overall syphilis/HIV co-infection rate in our study population was 2.2%. This co-infection rate is about two to four times higher than the rate recently reported among pregnant women in Gondar [5, 6]. This calls for strengthening the integration of syphilis screening in the established antenatal HIV prevention programs [31] and implementation of rapid dual HIV/syphilis screening test in ANC clinics in Ethiopia to improve syphilis and HIV screening coverage, early detection, and maternal treatment to prevent infant infection [9, 30].

According to WHO, delayed antenatal care (after the 12th gestational week) is one of the barriers for the control of adverse pregnancy outcomes [14]. It is associated with fewer medical consultations and fewer routine examinations particularly in resource limited countries like Ethiopia where non-specific (RPR) test is widely used. The test may not indicate syphilis diagnosis in a single visit and perhaps missed the positive cases particularly in the presence of concurrent HIV infection. In the present study, delayed antenatal care was observed in 60.3% of pregnant women in the first ANC visit. It is an indicative that many pregnant women may have been diagnosed and treated after the recommended gestational age and are more likely to attain poor outcomes of pregnancy [14]. Additionally, high seroprevalence of syphilis in those women who visited ANC services more than once and women at third trimester suggests that those women visited the clinic more than once may either not properly screened or treated in their first ANC visit. Taken together, this observation highlights the need to strengthen the existing ANC services to reduce mother to child transmission of syphilis [11].

Nevertheless, sample size limited our ability to show significance for many demographic variables. We did not evaluate birth outcomes of mothers treated for syphilis due to the cross sectional nature of the study. We did not also report on syphilis screening and treatment coverage within pregnant women seen at this facility overall. Regardless of the depicted limitations, this study ultimately adds supportive information on increased prevalence of syphilis in the study area.

## Conclusions

The seroprevalence of syphilis among pregnant women in this hospital in Southern Ethiopia was considerably higher than in previous studies in Ethiopia. Women with HIV infection were significantly more likely to be syphilis-seropositive. Syphilis/HIV co-infection coupled with other gaps in the health care, such as lack of specific diagnostic tests and delayed ANC visits in Ethiopia; contribute to the persistence of syphilis as a major public health problem. Therefore, it is necessary to design strategies for health professionals in order to assure the minimum number of visits required by the Ethiopian Ministry of Health. In addition, timely access to dual detection of HIV and syphilis and treatment for those tested positive for syphilis should be of extreme concern and priority in the control of syphilis. Furthermore, associated factors for syphilis transmission need to be further studied in large sample size in different health centre in the region and in the country at large, which will be helpful to tailor the current health education programs at antenatal care clinics to raise the awareness of mothers.

## Abbreviations

ANC: Antenatal care
ART: Antiretroviral therapy
HIV: Human immunodeficiency virus
RPR: Rapid plasma regain
SNNPR: Southern Nations and Nationalities People’s Region
SSA: Sub-Saharan Africa
TPHA: *Treponema pallidum* haemagglutination assay
WHO: World Health Organization
